# Preliminary expression profile of cytokines in brain tissue of BALB/c mice with *Angiostrongylus cantonensis* infection

**DOI:** 10.1186/s13071-015-0939-6

**Published:** 2015-06-14

**Authors:** Liping Yu, Xiaoying Wu, Jie Wei, Qi Liao, Lian Xu, Siqi Luo, Xin Zeng, Yi Zhao, Zhiyue Lv, Zhongdao Wu

**Affiliations:** Department of Preventive Medicine, School of Medicine, Three Gorges University, Yichang, China; Department of Parasitology, Zhongshan School of Medicine, Sun Yat-sen University, Guangzhou, China; Key Laboratory for Tropical Diseases Control, The Ministry of Education, Sun Yat-sen University, Guangzhou, China; Department of Preventive Medicine, School of Medicine, Ningbo University, Ningbo, China; Advanced Computing Research Laboratory, Institute of Computing Technology, Chinese Academy of Sciences, Beijing, China

**Keywords:** *Angiostrongylus cantonensis*, Eosinophil, Mouse model, Cytokine, Chemokine

## Abstract

**Background:**

*Angiostrongylus cantonensis* (*A. cantonensis*) infection can result in increased risk of eosinophilic meningitis. Accumulation of eosinophils and inflammation can result in the *A. cantonensis* infection playing an important role in brain tissue injury during this pathological process. However, underlying mechanisms regarding the transcriptomic responses during brain tissue injury caused by *A. cantonensis* infection are yet to be elucidated. This study is aimed at identifying some genomic and transcriptomic factors influencing the accumulation of eosinophils and inflammation in the mouse brain infected with *A. cantonensis*.

**Methods:**

An infected mouse model was prepared based on our laboratory experimental process, and then the mouse brain RNA Libraries were constructed for deep Sequencing with Illumina Genome Analyzer. The raw data was processed with a bioinformatics’ pipeline including Refseq genes expression analysis using cufflinks, annotation and classification of RNAs, lncRNA prediction as well as analysis of co-expression network. The analysis of Refseq data provides the measure of the presence and prevalence of transcripts from known and previously unknown genes.

**Results:**

This study showed that Cys-Cys (CC) type chemokines such as CCL2, CCL8, CCL1, CCL24, CCL11, CCL7, CCL12 and CCL5 were elevated significantly at the late phase of infection. The up-regulation of CCL2 indicated that the worm of *A. cantonensis* had migrated into the mouse brain at an early infection phase. CCL2 could be induced in the brain injury during migration and CCL2 might play a major role in the neuropathic pain caused by *A. cantonensis* infection. The up-regulated expression of IL-4, IL-5, IL-10, and IL-13 showed Th2 cell predominance in immunopathological reactions at late infection phase in response to infection by *A. cantonensis*. These different cytokines can modulate and inhibit each other and function as a network with the specific potential to drive brain eosinophilic inflammation. The increase of ATF-3 expression at 21 dpi suggested the injury of neuronal cells at late phase of infection. 1217 new potential lncRNA were candidates of interest for further research.

**Conclusions:**

These cytokine networks play an important role in the development of central nervous system inflammation caused by *A. cantonensis* infection.

**Electronic supplementary material:**

The online version of this article (doi:10.1186/s13071-015-0939-6) contains supplementary material, which is available to authorized users.

## Background

*Angiostrongylus cantonensis* (*A. cantonensis*) is a parasitic nematode that causes human angiostrongyliasis, the most common cause of human esoinophilic meningitis (EM) in Southeast Asia and the Pacific Basin. In recent years, more cases and several outbreaks of the disease have been reported in endemic regions [1–4]. The most common clinical manifestations of EM are fever, bitemporal or frontal headache, painful paresthesias, stiff neck and vomiting [5, 6]. Previous studies have shown that the invasion of the central nervous system (CNS) by developing larvae of the parasite can cause eosinophilic inflammation [7–9]. The larvae generally remain in the CNS and eosinophil recruitment can cause neuropathological damage [10–13].

Inflammatory factors play a key role in the development of brain inflammation. Cytokines are a broad category of small proteins released by a large range of cells including chemokines, interferons, interleukins, tumor necrosis factor, growth factor and colony stimulating factor [14]. It has been shown that the CC type chemokines are involved in mediating eosinophil chemotaxis in various allergic inflammatory responses and autoimmune diseases through interaction with corresponding receptors on the surface of eosinophils [15–19]. The CC type of chemokines consists of at least 28 members (CCL1-28). Studies have shown a critical role for chemokines CCL 2, CCL 3, CCL 5, CCL 7, CCL 11, CCL 12, CCL 24 and CCL 28. Among these CCLs, CCL-11 (CSF eotaxin) has been shown to be associated with eosinophilic meningitis in angiostrongyliasis. Previous studies have demonstrated that increased levels of the (CCL 11) correlated with CSF eosinophilia in both clinical patients and animal models infected with *A. cantonensis* [11, 20]. In our previous study, these findings have also been verified [21]. However, the involvement of chemokines and interleukins in the inflammatory processes caused by *A. cantonensis* infection was not completely clear. The underlying mechanism of the accumulation of eosinophils and the regulation of associated molecules has yet to be investigated. Currently there are very few reagents that are sensitive enough for early diagnosis and there are very few therapeutic drugs for early treatment. A better understanding of the inflammatory process in the CNS and associated molecular mechanisms caused by this parasite will provide some valuable insight for the development of possible novel diagnostic and therapeutic agents.

In our present study, the EM animal model with *A. cantonensis* infection was prepared using Balb/C mice, the transcriptome analysis of the mouse brain was done using RNA-seq by Illumina sequencing, the objective is to investigate the transcript dysregulation related to inflammatory processes caused by *A. cantonensis* infection.

## Methods

### Ethics statement

Animals were cared for in accordance with the guidelines developed by the China Council on Animal care, and all animal experiments were performed according to the procedures approved by the Animal Care and Use Committee of Guangdong Province, China.

### Preparation of animal infection model

The third-stage larvae of *A.cantonensis* were obtained from the infected Amazonian snail (*Ampullaria gigas*) by digestion of the snail tissue in artificial gastric juice (0.3 % pepsin, 0.7 % HCL and 10 ml/g tissue) at 37 °C for 2 h. The precipitates were then washed with distilled water, and the third-stage larvae were counted and collected by microscopy [22].

Fifteen Balb/C mice weighing 18-20 g were purchased from the centre of Experimental Animals, Sun Yet-Sen University. All animals were kept in a germ-free environment with free access to standard pellet diet and clean water. They were divided into 5 groups (infection for 2 days, 7 days, 14 days, 21 days and normal control group). All the treatment groups were infected with 30 ± 2 *A.cantonensis* by intragastric administration. Another 25 Balb/C mice of the same standard were also divided into 5 groups to prepare samples for Q RT-PCR.

### Sample collection and RNA Extraction

In this study, the mouse brain tissues were collected on the 2nd, 7th, 14th and 21st day post-infection. Similarly the samples from the control mice were collected on the 21st day post infection, and the samples were frozen in liquid nitrogen. Three samples were collected separately to prepare five pools representing an infection model and control mice for constructing RNA libraries. Total RNA was extracted using Trizol reagent (Invitrogen, Carlsbad, CA, USA) according to the manufacturer’s instructions. After total RNA was resuspended in DEPC-treated water, they were stored at −80 °C until further use. The quantity and integrity of the total RNA was assessed with an Agilent 2100 Bioanalyzer (Agilent Technologies, USA).

### Histochemistry examination

Samples were collected at 21 dpi (days post infection). Blood samples were drawn from the tail of the control and EM mice from 9 to 11 in the morning and used to make peripheral blood smears, followed by staining using the Wright Giemsa staining method for the detection of eosinophils. Specimens of brain tissue of these two groups were fixed in 4 % paraformaldehyde for 2 days. Then they were embedded in paraffin, serially sectioned and stained with hematoxylin eosin (HE) according to the conventional staining methods [23].

### Construction of RNA libraries and deep sequencing

The RNA libraries were constructed from five groups respectively. The overall flow of RNA library construction and deep sequencing is shown schematically in Additional file 1: Figure S1. In brief, isolation and purification of mRNA, conversion of RNA to cDNA, followed by addition of sequencing adapters. Subsequently, the ligated RNAs were used as templates for RT-PCR amplification. The DNA sequencing was performed with Illumina Genome Analyzer to produce digital-quality data after the purification of the PCR products (Additional file 1: Figure S1). Those genes that were regulated more than 2-fold and p < 0.05 were considered as differentially expressed [24]. The expression levels of transcripts were calculated using Cufflinks, and the data was processed with R software.

The raw data was processed with a bioinformatics’ pipeline as follows: (1) Filter low quality tags; (2) Trim adaptor; (3) Clean reads mapping to the *Mus musculus* 9 genome using TopHat; (4) Refseq genes expression analysis using cufflinks; (5) Annotate and classify RNAs into different categories; (6) LncRNA prediction; (7) Co-expression network (Additional file 2: Figure S2).

LncRNA prediction TopHat was used to blast the sequences with the reference genome. Then the transcripts were re-built with Cufflinks. These transcripts were compared to previously annotated genes with Cuffcompare and obtained the information about the intergenic transcript, full intron transcript and antisense transcript. These new transcripts were used to further lncRNA prediction. According to new Transcript length > 200 bp, new transcript ORF (Open Reading Frame) length < 300 and characters of code and noncode genes of known database, noncode screening model was built to screen new lncRNA [25]. Cufflinks calculated the expression of these new lncRNA, and Cuffdiff analyzed the differential expression of them.

### Analysis of refseq genes Co-expression network

After removing the transcripts (with the maximum FPKM from 5 samples < 1), expression data was normalized. By applying the variance filters, the low variance (one-fourth) was excluded from the total refseq genes. Then the Pearson correlations were calculated for all pairs of refseq genes left and used as the basis for building the networks. We calculated the Fisher’s asymptotic P values using WGCNA program of R. Then the multtest of R was used to adjust the P values into q values with BH multitest adjustments. The data of the top former and latest 0.5 % correlations with q < 0.01 were kept to build the refseq genes co-expression network. The hub-gene function analysis: For each non-coding gene, GO annotation for each code gene which was co-expressed with the non-coding gene (with p < 0.05, the number of co-expressed mRNA ≥ 2). Function analysis of module: Markov Cluster Algorithm (MCL) method was used for mining the module of the network, and GO annotation for code genes of each module (with p < 0.05, the number of code gene of each mode ≥ 2) [26].

### Quantitative RT-PCR

Quantitative RT-PCR was used to verify the CCL type gene expression. The cDNA synthesis of these genes was prepared using First-Strand cDNA Synthesis Kit (Invitrogen) following the manufacturer’s protocols. Then the RT-PCR was performed with SYBR Green Supermix (BioRad) according to the protocol as follows: (1) 95 °C for 2 min; (2) 40 cycles of 95 °C for 15 s, 60 °C for 15 s and 72 °C for 15 s; (3) melting curve analysis; (4) 50 °C for 30s; (4) All reactions were run in triplicate. These primers were synthesized by Life Technologies Company and the actin beta gene was used as control (Additional file 3: Table S1). The expression levels of these genes were measured by Ct value (threshhold cycle). And the relative expression level was calculated with the equation 2^-ΔΔCt^.

## Results

### Inflammation and pathological injuries of the brain tissue of infected mice

**Fig. 1:**
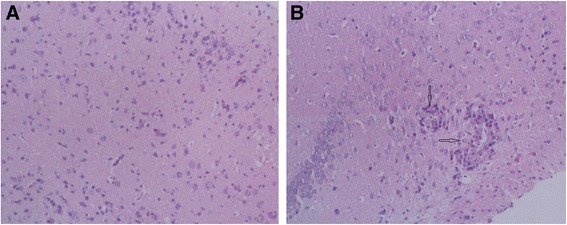
HE staining of the brain tissues taken from control (**a**) and *A. cantonensis* infection group of 21 days (**b**). A: ×100. B: ×100, pathological injuries showing focal necrosis and infiltration of inflammatory cell in the brain tissue (showed by black star)

The relative eosinophil count of white blood cells in peripheral blood of mice on 21st day post-infection with *A.cantonensis* was 2.67 ± 0.58, which was significantly higher than the control group 1.33 ± 0.58 (p < 0.05). HE staining showed that pathological injuries in brain parenchyma of the infected mice included hemorrhages, dilated vessels, necrosis and infiltration of a large amount of inflammatory cells (Fig. 1). These findings indicated that the infected mouse model was constructed successfully.

### Gene-expression analysis

A total of 37.87G reads were generated from mouse brain tissue, of them 8.13G, 8.18G, 7.90G, 7.57G and 6.09G for post-infection of 2 days, 7 days, 14 days, 21 days and control groups respectively. After filtering out contaminating reads, a total of 149,556,822 reads with an average map rate of 63.24 % were produced from mouse brain cDNA libraries. The clean reads were assembled and blast with the reference genome using TopHat. Then Cufflinks generated a total of 24,942 transcripts.

The expression of IL-4 and IL-13 in brain tissue improved significantly at 14 dpi and 21 dpi. IL-19, IL-10, IL-6, IL-27 and IL-5 also increased during late infection days. Besides these IL-1b, IL-15 and IL-1a also increased more than 5 fold at 21 dpi (Table 1). The expression of ATF-3 improved significantly at late infection phase (Additional file 4: Table S2).

**Table 1:** Differential expression of interleukin. FPKM values of interleukin from brain tissue for different infection days (2 days, 7 days, 14 days and 21 days) in comparison with control group are listed

Gene Symbol	Con	2 days	7 days	14 days	21 days
Il10	0	0	0	0.188	0.378
Il11	0.247	0.182	0.353	0.437	0.458
Il13	0	0	0	0.979	1.644
Il15	0.042	0.223	0	0.618	0.319
Il16	2.608	2.831	13.538	1.240	1.255
Il18	17.449	15.415	9.336	20.460	13.901
Il19	0	0.042	0	0.044	0.746
Il1a	0.151	0.358	0.058	1.640	0.904
Il1b	0.359	0.176	0.095	6.454	8.003
Il1f5	0	0	0.250	0	0
Il22	0.157	0.038	2.725	0.091	0.049
Il23a	0.270	0.342	0.825	0.506	0.215
Il25	0.379	0.278	0.128	0.250	0.101
Il27	0	0	0	0.081	0.295
Il3	0	0	0	0.118	0.000
Il33	3.378	2.590	1.275	3.280	2.398
Il34	18.909	18.201	11.212	14.285	20.597
Il4	0	0	0	0.238	2.600
Il5	0	0.024	0	0.178	0.276
Il6	0	0	0	0.202	0.341
Ilf2	34.311	32.975	39.354	36.748	33.950
Ilf3	5.945	4.882	5.886	4.881	4.977

CCL2 (MIP-1) was not secreted in the control group, its expression improved at 2 dpi and increased to the highest point at 21 dpi. CCL2 had the most significant change at 14 dpi and 21 dpi of the CC type chemokine expression data. It was followed by the change of expression of CCL8 at 14 dpi and 21 dpi. CCL1 (I-309), CCL24 (Eotaxin-2), CCL11 (Eotaxin), CCL9 (MIP-3β), CCL6, CCL7 (MCP-3), CCL12 and CCL4 (MIP-1β) were increased more than 50 fold at 21 dpi compared to the control group. CCL5 showed the highest value at 14 dpi (Table 2).

**Table 2:** Differential expression of CC type chemokines. FPKM values of CC type chemokines from five groups represented their expression level

Gene Symbol	Con	2 days	7 days	14 days	21 days
Ccl21a	0.277	0.049	0.709	1.077	0.463
Ccl1	0	0	0	0.342	3.953
Ccl11	0	0	0	2.760	3.329
Ccl12	1.520	2.547	2.537	225.273	146.464
Ccl17	9.118	7.288	4.203	16.460	16.052
Ccl2	0	0.181	0.581	19.173	28.350
Ccl22	0.134	0.139	0.256	4.785	1.473
Ccl25	1.246	1.197	1.018	1.851	1.935
Ccl27a	10.451	22.708	48.381	31.936	18.279
Ccl3	0.775	1.063	1.001	5.904	9.697
Ccl4	0.598	1.806	0.549	9.863	34.128
Ccl5	0.779	0.548	3.889	41.466	27.276
Ccl6	0.899	0.530	0.513	103.572	161.367
Ccl7	0.253	0	0	22.451	38.048
Ccl8	0.646	0.453	1.895	1021.010	1573.200
Ccl9	0.149	0.243	0.071	27.302	41.139
Ccl19	0.246	0.238	0.469	1.495	0.831
Ccl24	0.290	0.488	0.590	41.561	109.315
Ccl27b	4.194	4.018	6.907	6.177	3.441
Ccl21b	0.033	0.061	0.092	0.110	0.210

Some lncRNAs showed significantly altered expression in infected brain tissues at the late phase of infection. H19 improved significantly at 14 dpi. Malat1 increased nearly 3 fold at late infection phase (Additional file 4: Table S2).

### Quantitative RT-PCR

**Fig. 2:**
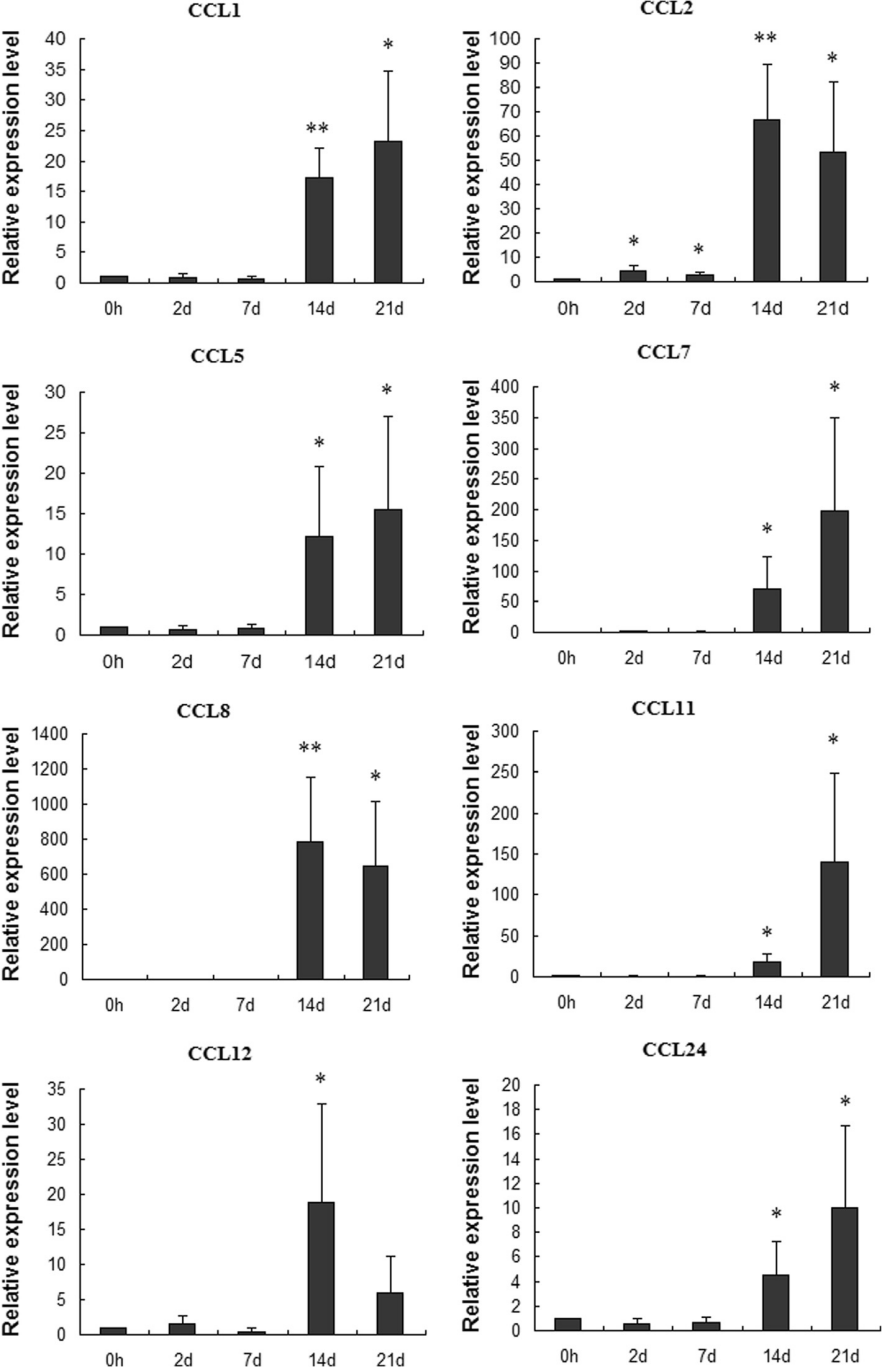
Differential expression of CC chemokines validated by Q RT-PCR. The relative expression level was calculated with the equation 2^-ΔΔCt^ and the error bars represented the standard error (n = 5). * represents p < 0.05, and **represents p < 0.01 compared with control group

To validate the deep sequencing data, the expression levels of CCL1, CCL2, CCL5, CCL7, CCL8, CCL11, CCL12, CCL24 were selected and quantified using Quantitative RT-PCR. The results showed that CCL1, 5, 7, 11, 24 secretion were increased with the development of CNS inflammatory progression. These CC type chemokines had relatively lower expression at 7 dpi, and increased to peak at 21 dpi. The expression level of CCL2 increased significantly at the early infection phase and reached the peak at 14 dpi and still showed high expression at the late infection phase. CCL 8 and 12 were found to increase and reached the peak value at 14 dpi and also had higher expression at 21 dpi (Fig. 2). The results showed that these differentially expressed chemokines were involved in CNS inflammatory progression.

### LncRNA prediction

Cufflinks regenerated transcripts. These transcripts were compared to previously annotated genes with Cuffcompare. Applying the screening model, a total of 1217 new lncRNA were obtained (Additional file 5: Table S3). The expression of these putative lncRNAs were analysed and it was shown that they were mainly distributed in chr2, chr11 and chr1 separately 158, 155 and 147 type (Additional file 6: Table S4, Table 3). These lncRNA candidates’ data analyses will help to guide further lncRNA identifications and characterizations.

**Table 3:** New lncRNA number of different chromosome

Chromosome	Num
chr1	147
chr10	75
chr11	155
chr12	95
chr13	76
chr14	79
chr15	93
chr16	79
chr17	103
chr18	63
chr19	53
chr2	158
chr3	41

### Co-expression network

**Fig. 3:**
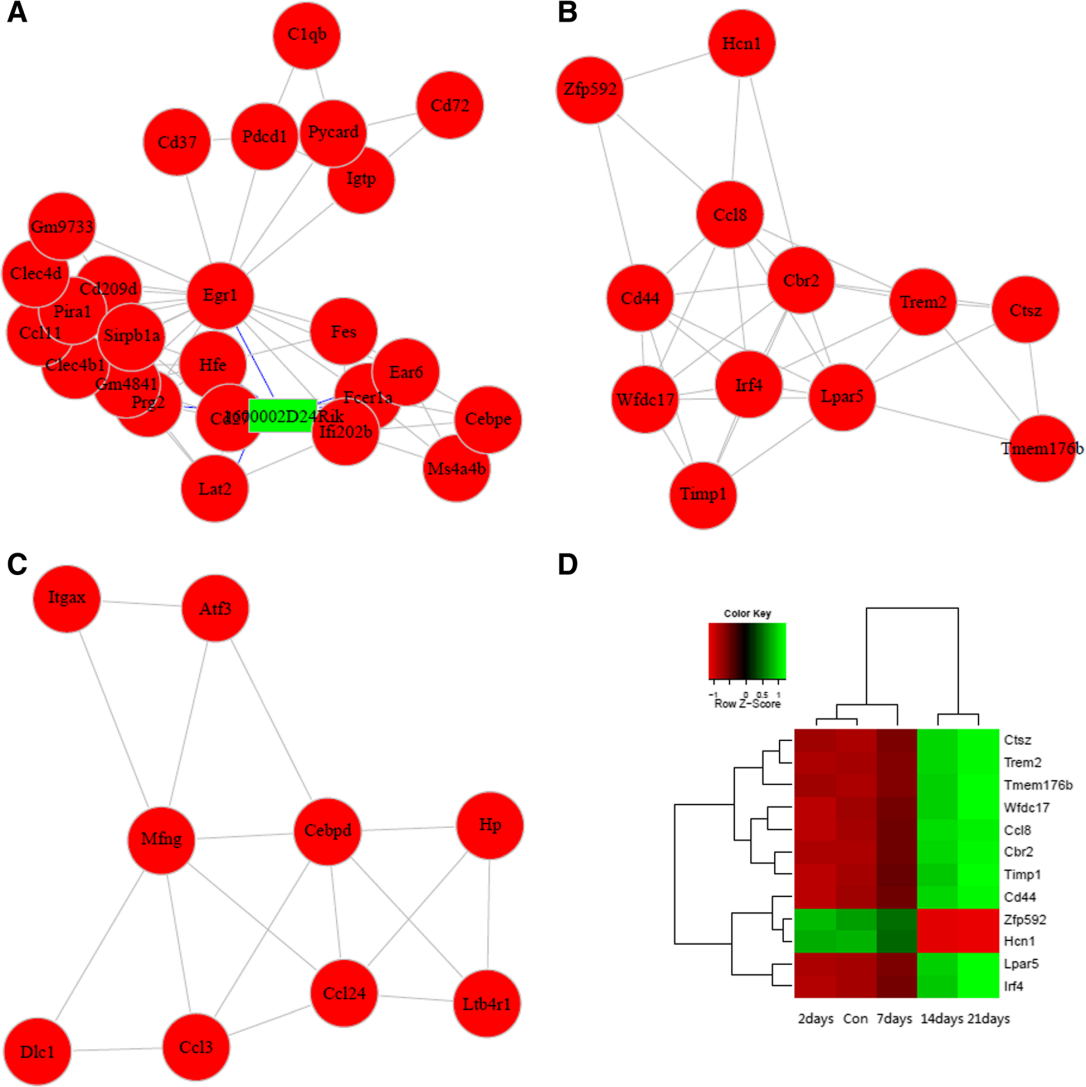
Module analysis results (partial). (**a**) net of module including CCL11, (**b**) net of module including CCL8, (**c**) net of module including CCL24, (**d**) Cluster analysis of genes of module including CCL8

**Fig. 4:**
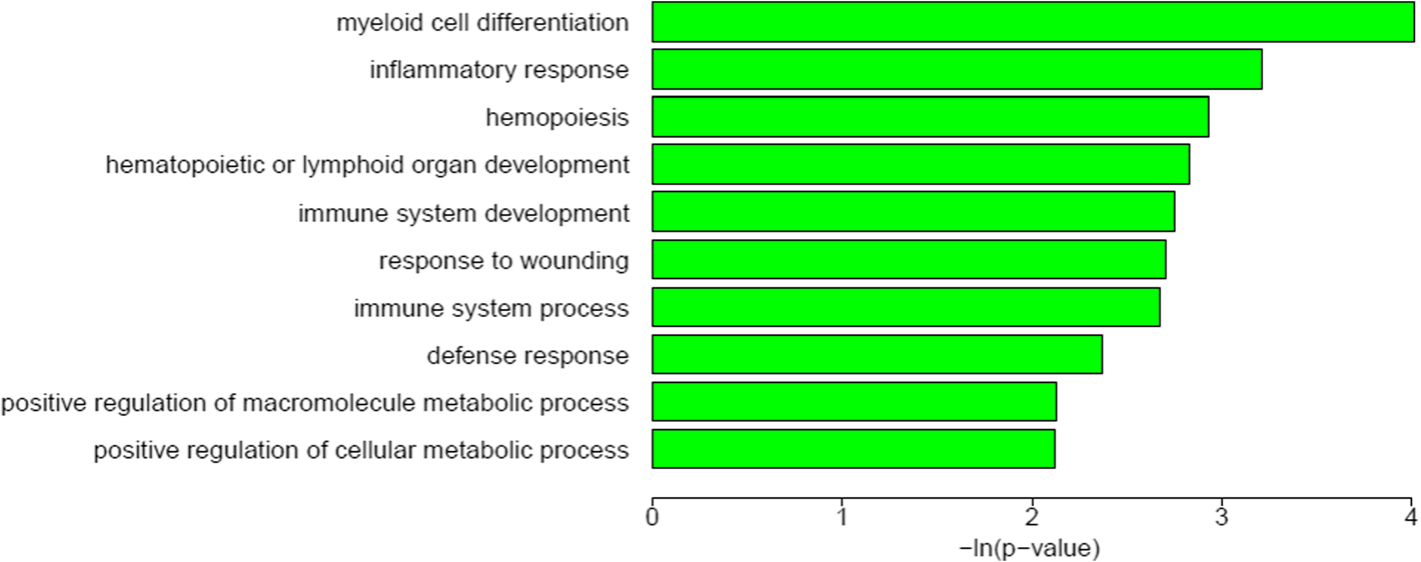
GO enrichment analysis (partial). GO enrichment analysis from biological process for the code genes of the module including CCL8

Weighted gene co-expression network analysis identified 180 co-expression modules which include the number of code gene ≥ 8. Ccl3, Ltb4r1, Cebpd, Mfng and Hp were highly and significantly correlated with the expression of CCL24. Gm4841, Clec4b1, Sirpb1a, Cd209d, Clec4d, Pira1, Egr1, Prg2 and Gm9733 were also highly and significantly correlated with the expression of CCL11. Significant correlation was shown in Zfp592, Wfdc17, Trem2, Cd44, Lpar5, Hcn1, Cbr2 and Irf4 with CCL8 (Fig. 3). The expression of CCL8 is correlated significantly with CCR8. The GO enrichment analysis from biological process of this module revealed that the two most enriched GO terms were myeloid cell differentiation and inflammatory response (Fig. 4). The co-expression network analysis showed that ATF-3 was highly and significantly correlated with the expression of Cebpd and Itgax (Additional file 7: Table S5).

## Discussion

Our former research showed CCL8 and CCL11 in mouse brain samples increased gradually in parallel with the rising eosinophil counts in blood after infection [21]. In this study, these CC type chemokines (CCL1, 5, 7, 11, 24) normally had no or less secretion, however, they improved significantly at 14 dpi and reached the top at 21 dpi. This phenomenon is consistent with the eosinophil change trend in cerebrospinal fluid (CSF) and peripheral blood. It has been shown that the worm recovery rate increased along with the infection time, highest at the third week in the infected mouse brain [27]. These chemokines serve as mediators for eosinophil recruitment in the development of CNS inflammation caused by *A. cantonensis* infection.

The expression of CCL2 also improved significantly during the early infection phase. It has been demonstrated that CCL2 could be induced in the brain during injury and trauma [28–30]. Research showed that the larvae migrated to the host brain tissue in the second day after infection. The movement of the larvae induced the injury of brain neurons [27]. The neurons and astrocyte can release the CCL2 in the pathogen stimulation [31]. Therefore, the invasion of *A. cantonensis* may induce some inflammatory factors such as CCL2 secretion in the brain at early infection. The involvement of CCL2 in neuropathic pain processing has been established in animal models [32, 33]. This up-regulation of CCL2 correlated with local monocyte and macrophage infiltration and pain processing [34, 35]. CCL2 might play a major role in the neuropathic pain caused by *A. cantonensis* infection.

CCL8 showed significant changes at 14 dpi and 21 dpi. Module function analysis of co-expression network revealed that Lpar5 was highly and significantly correlated with the expression of CCL8. Lpar5 was one of the five known G protein-coupled receptors (GPCRs) [36]. GO analysis showed this module was mainly focused in the inflammatory response. In this study, CCR8 also increased at the late infection phase and its expression is correlated significantly with CCR8. CCR8-CCL8 is a newly identified chemokine receptor-ligand pair that mediates the skin accumulation of TH2 cells with the specific potential to drive chronic eosinophilic inflammation [37]. These data indicated that CCR8-CCL8 might be involved in recruitment of Th2 cells during the development of CNS inflammation caused by *A. cantonensis* infection.

These chemokines such as CCL2, 5, 7 and 8 played a key role in the specific recruitment of Th2 cells and eosinophils [38–40]. According to previous research, Th2-type cytokines may also stimulate recruitment of eosinophils and neutrophils through the release of CC (RANTES, MCP-3 and −4, eotaxin and eotaxin-2) and CXC chemokines [41]. In this current study, IL-4, IL-13, IL-19, IL-10, IL-6, IL-27 and IL-5 showed almost no expression in the normal control group, whilst at 14 dpi and 21 dpi, IL-4 and IL-13 significantly increased, followed by IL-19, IL-10, IL-6, IL-27 and IL-5, which were highly expressed. The migration of the worm in the brain of host induced injury and inflammation of CNS [42, 43]. Macrophages, eosinophils and lymphocytes gathered at the site of inflammation and Th2-type cytokine secretion such as IL-4, IL-5, IL-10, and IL-13 were increased under the stimulation of worm antigen in the late infection phase. These might also promote the secretion of CC type chemokines. IL-5 plays a major role in the regulation of eosinophil formation, maturation, recruitment and survival [44]. Its improvement correlated with the trend of worm recovery in the brain of mice and the count of eosinophils, which also showed significant improvement both in peripheral blood and CSF at the late infection phase. IL-6 is primarily produced at sites of acute and chronic inflammation, it functions in inflammation and the maturation of B cells, and chronically elevated IL-6 levels lead to chronic inflammation and fibrotic disorders [45]. IL-19 belongs to the IL-10 cytokine subfamily and it can lead to the activation of the signal transducer and activator of transcription 3 (STAT3) [46]. It was reported that it can lead to the higher expression of IL-6 and TNF-alpha and induce apoptosis which suggested it was involved in inflammatory responses and promoted the development of CNS inflammation [47–49]. There can be interplay between these different cytokines that can modulate cytokine levels and either up regulate or inhibit each other and function as a network with the specific potential to drive brain eosinophilic inflammation.

The pathological process induced by inflammatory factors in CNS has also been associated with changes in the transcription factor ATF-3, which plays a role in regulating axon growth and regeneration, and the induction of ATF-3 is linked with injury of the axon [50]. The pattern of its gradually increasing was in accordance with variation of the number change of worm body and pathological changes in the mouse brain. This indicated that the migration of the worm into the brain caused the injury of nerve cell and induced the high expression of ATF-3.

Recent studies have highlighted the roles of long non-coding RNAs (lncRNAs) in cancer, cardiovascular disease and other inflammation related diseases, and it is suggested that they might play critical roles in inflammation progression [51–53]. H19 genes are imprinted in mammals [54]; it showed different expression during late *A. cantonensis* infection. 1217 putative lncRNA were obtained in this study. According to the analysis by co-expression modules, it was shown that some lncRNAs was highly and significantly correlated with the expression of CCL24, CCL11and CCL8. Those *A. cantonensis* infection-related lncRNAs may provide potential novel therapeutic targets for *A. cantonensis* infection and other diseases. At present, we are preparing the animal and cell experiments to identify the checkpoint correlated to CCL8.

## Conclusions

These cytokine networks play an important role in the development of CNS inflammation caused by *A. cantonensis* infection.

## Additional files

Additional file 1: Figure S1. The overall flow of RNA library construction and deep sequencing. Additional file 2: Figure S2. The overall flow of analysis of sequence data. Additional file 3: Table S1. Primers of CC chemokines used in Q RT-PCR. Additional file 4: Table S2. All Refseq genes’ expression. Additional file 5: Table S3. New lncRNA information. Additional file 6: Table S4. New lncRNA’s expression. Additional file 7: Table S5. The co-expression net-work.
