# Supplementary figures and images for: Preliminary expression profile of cytokines in brain tissue of BALB/c mice with Angiostrongylus cantonensis infection
Source: Parasit Vectors. 2015 Jun 14;8:328. doi: 10.1186/s13071-015-0939-6 (PMC4476182; doi:10.1186/s13071-015-0939-6)

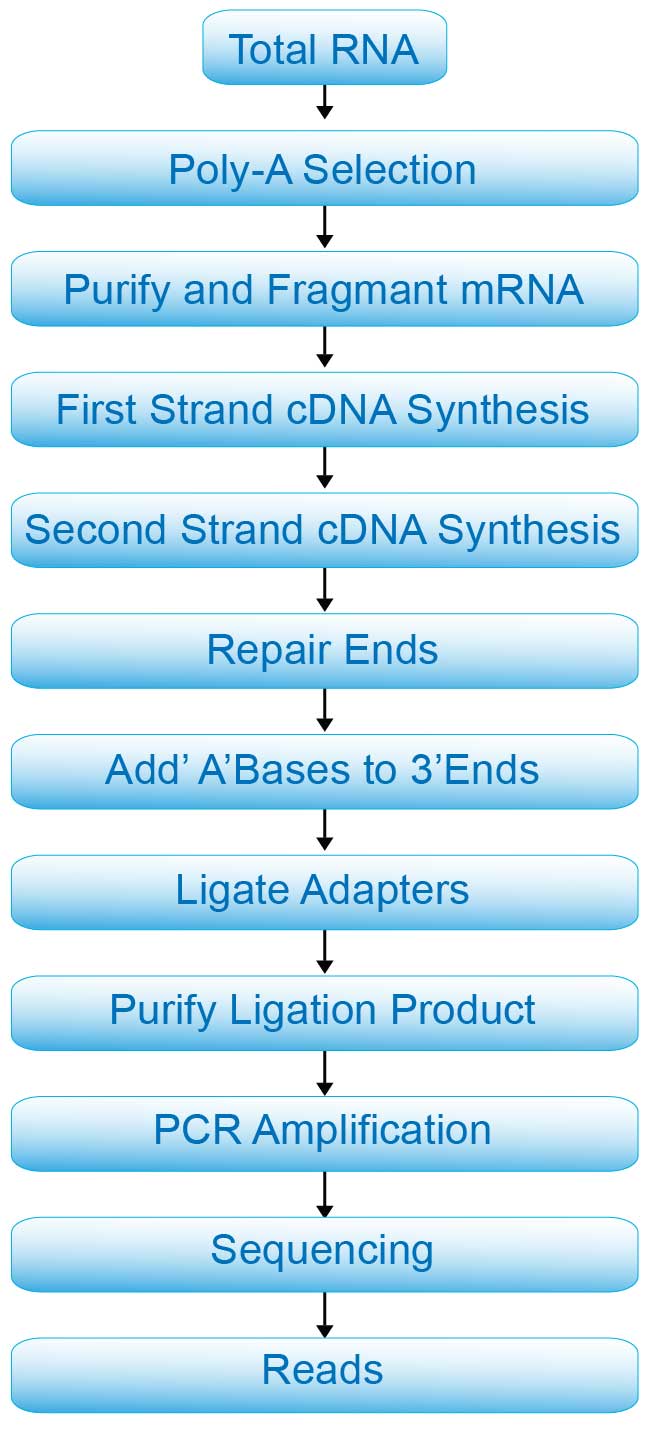

Supplement: Additional file 1: Figure S1. — The overall flow of RNA library construction and deep sequencing. [file 13071_2015_939_MOESM1_ESM.jpg]

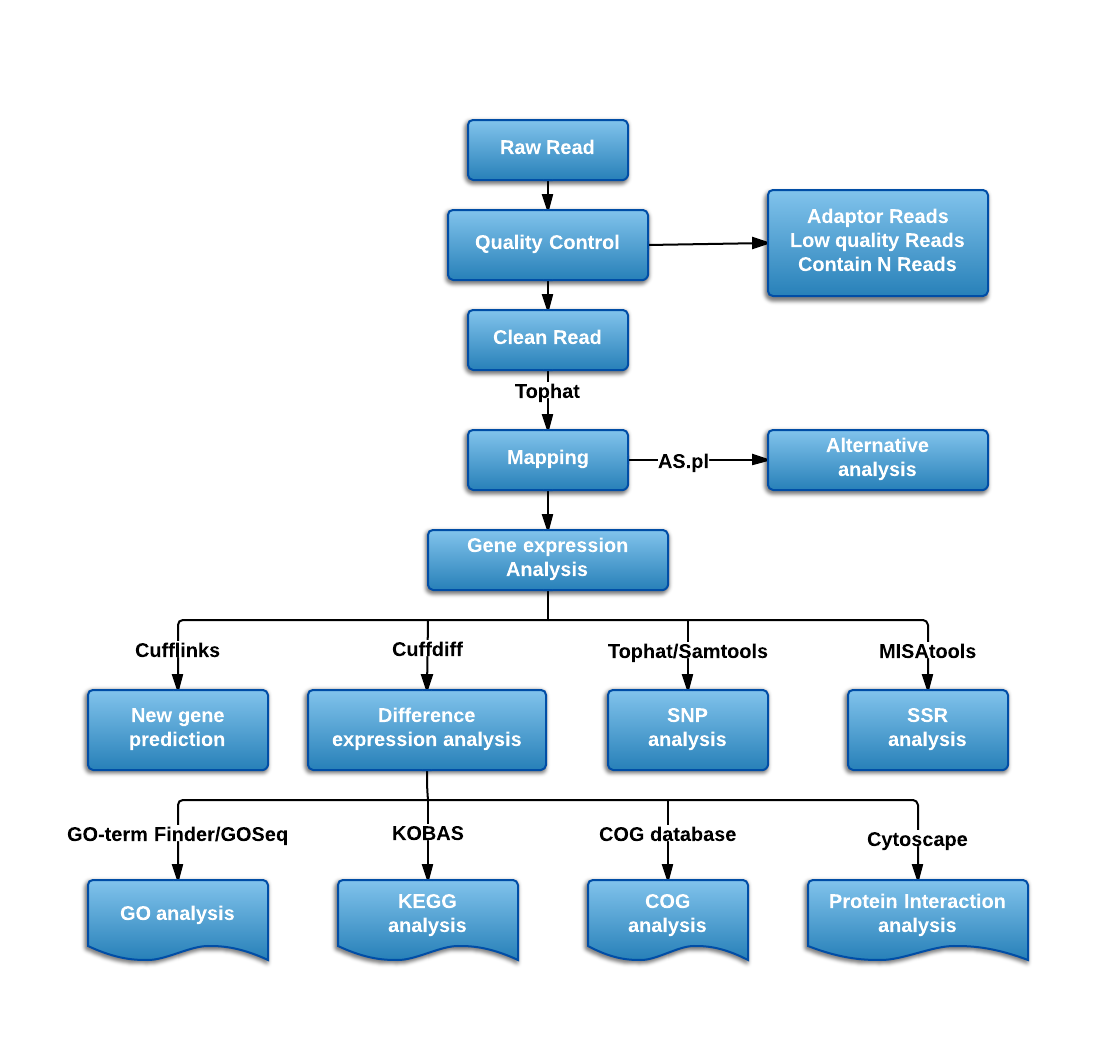

Supplement: Additional file 2: Figure S2. — The overall flow of analysis of sequence data. [file 13071_2015_939_MOESM2_ESM.png]
